# AMPK Profiling in Rodent and Human Pancreatic Beta-Cells under Nutrient-Rich Metabolic Stress

**DOI:** 10.3390/ijms21113982

**Published:** 2020-06-01

**Authors:** Thierry Brun, Cecilia Jiménez-Sánchez, Jesper Grud Skat Madsen, Noushin Hadadi, Dominique Duhamel, Clarissa Bartley, Lucie Oberhauser, Mirko Trajkovski, Susanne Mandrup, Pierre Maechler

**Affiliations:** 1 Department of Cell Physiology and Metabolism & Faculty Diabetes Center, University of Geneva Medical Center, 1206 Geneva, Switzerland; Thierry.Brun@unige.ch (T.B.); Cecilia.Jimenez-Sanchez@unige.ch (C.J.-S.); noushin.hadadi@unige.ch (N.H.); Dominique.Duhamel@unige.ch (D.D.); Clarissa.Bartley@unige.ch (C.B.); Lucie.Oberhauser@unige.ch (L.O.); Mirko.Trajkovski@unige.ch (M.T.); 2 Functional Genomics and Metabolism Research Unit, Department of Biochemistry and Molecular Biology, University of Southern Denmark, 5230 Odense, Denmark; jgsm@bmb.sdu.dk (J.G.S.M.); s.mandrup@bmb.sdu.dk (S.M.)

**Keywords:** AMPK, ATP, fructose, pancreatic islets, beta-cell, insulin, glucotoxicity

## Abstract

Chronic exposure of pancreatic β-cells to elevated nutrient levels impairs their function and potentially induces apoptosis. Like in other cell types, AMPK is activated in β-cells under conditions of nutrient deprivation, while little is known on AMPK responses to metabolic stresses. Here, we first reviewed recent studies on the role of AMPK activation in β-cells. Then, we investigated the expression profile of AMPK pathways in β-cells following metabolic stresses. INS-1E β-cells and human islets were exposed for 3 days to glucose (5.5–25 mM), palmitate or oleate (0.4 mM), and fructose (5.5 mM). Following these treatments, we analyzed transcript levels of INS-1E β-cells by qRT-PCR and of human islets by RNA-Seq; with a special focus on AMPK-associated genes, such as the AMPK catalytic subunits α1 (*Prkaa1*) and α2 (*Prkaa2*). AMPKα and pAMPKα were also evaluated at the protein level by immunoblotting. Chronic exposure to the different metabolic stresses, known to alter glucose-stimulated insulin secretion, did not change AMPK expression, either in insulinoma cells or in human islets. Expression profile of the six AMPK subunits was marginally modified by the different diabetogenic conditions. However, the expression of some upstream kinases and downstream AMPK targets, including K-ATP channel subunits, exhibited stress-specific signatures. Interestingly, at the protein level, chronic fructose treatment favored fasting-like phenotype in human islets, as witnessed by AMPK activation. Collectively, previously published and present data indicate that, in the β-cell, AMPK activation might be implicated in the pre-diabetic state, potentially as a protective mechanism.

## 1. Introduction

### 1.1. AMPK and Glucose Homeostasis

At the cellular level, AMP-activated protein kinase (AMPK) is a sensor of the energy state, activated upon low ATP concentration. Translated at the level of the whole organism, the pancreatic β-cell is a sensor of energy intake, essentially decoded through blood glucose concentration. Following a meal, an increase in glycemia stimulates the secretion of insulin by the pancreatic β-cells located in the islets of Langerhans. Insulin induces blood glucose clearance, mostly by skeletal muscles, and inhibits hepatic glucose production. Accordingly, insulin regulates blood glucose through its action on its target tissues, promoting storage of metabolic substrates and energy homeostasis. The anabolic action of insulin favors storage of lipids and expansion of the adipose tissue. In the context of obesity, insulin becomes less efficient as a consequence of the development of insulin resistance by the target tissues. Such resistance can be counterbalanced by increased production of insulin by the pancreatic β-cells. As a consequence of overstimulation, the resulting workload pressure may induce various metabolic stresses on the β-cells, such as endoplasmic reticulum stress, mitochondrial dynamics alteration, oxidative stress, and β-cell dedifferentiation, as reviewed elsewhere [1]. Collectively, defects in the insulin-producing cells represent the triggering event leading to hyperglycemia that characterizes type 2 diabetes [2]. In these pathogenic conditions, AMPK pathways might play a role in the preservation of functional β-cells [3]. Growing evidence indicate that AMPK is involved in whole-body glucose homeostasis, potentially as a positive and negative modulator of insulin secretion from β-cells.

Intuitively, activation of AMPK in the β-cell should not accompany the stimulatory action of glucose on insulin secretion, the former resulting from low energy state and the latter being associated with robust ATP production. However, the numerous studies on these actions are somehow contradictory, as extensively reviewed by others [4,5]. Here, we will address the effects of metabolic stresses on the expression of the key components of the AMPK system.

### 1.2. Function of the Insulin-Secreting Cell

Pancreatic β-cells are neuroendocrine cells that are primarily stimulated by the most ubiquitous energy substrate, namely glucose. Other nutrients, such as fatty acids and amino acids, are not bona fide stimuli for the induction of insulin secretion, probably because they are recruited in the post-prandial state that should avoid elevation of circulating insulin in order to prevent hypoglycemia.

**Figure 1:**
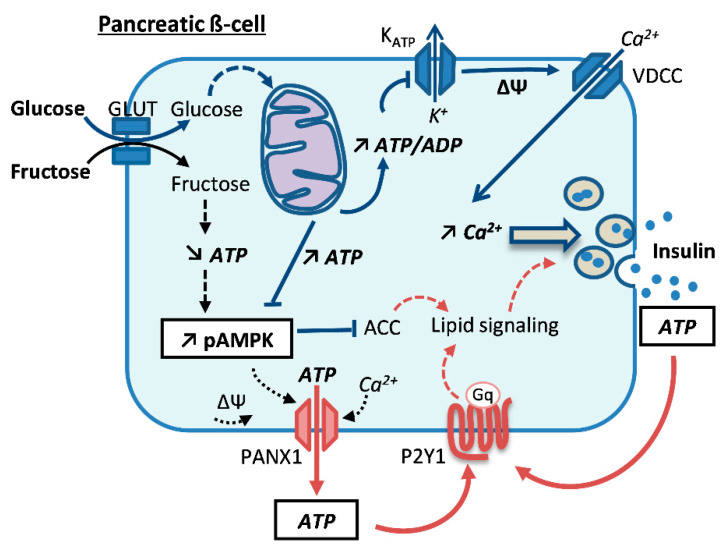
Regulation of insulin secretion by the pancreatic β-cell and intracellular/extracellular ATP signaling pathways. Glucose metabolism leads to the production of ATP in mitochondria. The ensuing elevation of the ATP/ADP ratio induces the closure of the K-ATP channels, which promotes the depolarization of the plasma membrane that opens the voltage-sensitive calcium channels. The resulting elevation of cytosolic calcium concentration triggers insulin release (blue arrows). Prolonged treatment with fructose induces a fasting-like phenotype in β-cells, resulting in AMPK activation even in the presence of glucose (black arrows). Fructose exposure activates Panx1 channels, promoting the release of cellular ATP and activation of purinergic P2Y1 receptors on the plasma membrane (red arrows). Chronic fructose exposure potentiates the stimulation of insulin secretion at intermediate glucose concentrations. Therefore, intracellular and extracellular ATP signaling pathways could play a concerted role in the β-cell as potentiators of glucose-induced insulin secretion.

Following a meal, glucose entering the β-cell activates a signal transduction cascade that must reflect the actual glycaemia in order to induce proportionate insulin release. Such a tight control relies on the production of intracellular signals, ensuring the coupling of glucose metabolism to insulin exocytosis [6]. Glucose-dependent generation of ATP by the mitochondria induces the closure of K-ATP channels on the plasma membrane, which promotes cell depolarization resulting in the opening of voltage-sensitive calcium channels. The ensuing increase in intracellular calcium triggers insulin exocytosis through a rapid first phase of secretion (Figure 1). Following this first transient phase, the production of additive factors amplifies the effects of calcium, resulting in a second sustained phase [7,8]. 

While glucose is the chief nutrient secretagogue of the β-cell, other metabolites, such as amino acids, fatty acids and fructose, may potentiate the effects of glucose on insulin secretion. The presence of some cell membrane components in the β-cells highlights the participation of specific signaling molecules, i.e., metabolites (fructose, glutamate, fatty acids) or nucleotides (ATP, ADP), as potent insulinotropic agents [9,10,11]. In this context, we recently reported that prolonged treatment with fructose enhances the secretory response of β-cells to physiological glucose concentrations [12]. Specifically, fructose exposure induces extracellular ATP signaling, resulting in potentiation of glucose-stimulated insulin secretion (Figure 1). This effect is mediated by activation of the purinergic P2Y1 receptor and is associated with the release of cellular ATP through pannexin-1 (Panx1) channels. Additionally, it is well documented that β-cells release ATP along with the exocytosis of insulin packed in the secretory granules. Regarding intracellular pathways, we reported that chronic fructose exposure induces AMPK activation in the β-cell [12], indicating that this sugar, as opposed to glucose, promotes a paradoxical intracellular fasting-like phenotype. Indeed, unlike glucose, the oxidation rate of fructose is not controlled by metabolic feedback following its uptake by cells expressing the sugar transporters GLUT5 and GLUT2. In hepatocytes, fructose is first converted to fructose-1-phosphate by fructokinase and further catabolized through an unregulated high-energy consuming pathway entering the second phase of glycolysis. This generates pyruvate and lactate and promotes fatty acid synthesis along with important intracellular ATP consumption. Of note, it remains uncertain whether physiological postprandial blood fructose levels could play a significant role in β-cell function, e.g., by modulating AMPK activation. 

### 1.3. AMPK in the β-Cell

The enzyme AMPK is a heterotrimeric complex composed of a catalytic α-subunit and two regulatory β- and γ-subunits. The AMPKβ subunit carries a carbohydrate-binding motif that mediates the recruitment of glycogen and the AMPKγ subunit contains the adenine nucleotide-binding site conferring the AMP, ADP, and ATP allosteric regulation of the AMPK trimer. Rodents and humans possess multiple isoforms of each subunit, encoded by different genes. Orthologs (α1, α2, β1, β2, γ1, γ2, γ3) of AMPK subunits are found in all eukaryotic species and are expressed in many tissues, suggesting that the structure and regulation of AMPK are evolutionarily conserved [13]. β-cells express multiple isoforms of the α, β and γ AMPK heterotrimer subunits, including both AMPKα1 and α2 proteins. In pancreatic β-cells, AMPK activity is controlled by glucose concentration [14]. Glucose-induced changes in AMPK activity are necessary and sufficient for the regulation of metabolic enzymes, such as pyruvate kinase (L-PK), at the transcriptional level [15]. AMPK also regulates expression of the K-ATP channel subunits KIR6.2 and SUR1. In response to sugar deprivation, channel synthesis rapidly increases by up-regulating translation of existing mRNAs [16]. Moreover, glucose deprivation modulates K-ATP channel trafficking at the plasma membrane, an effect mediated by AMPK [17]. These effects evoked by glucose deprivation are mimicked by pharmacological activation of AMPK using 5-aminoimidazole-4-carboxamide ribonucleotide (AICAR) and metformin. With lowering of the glucose levels, the activity of AMPK increases and, in parallel, insulin exocytosis slows down. This can be partially contributed by AMPK activation through the combined upregulation and translocation at the cell surface of K-ATP channel components. Indeed, increasing the number of channels at the cell membrane favors its hyperpolarization, thereby counteracting the insulin exocytosis. The β-cells which have lost their competence for glucose-induced insulin secretion do not exhibit such changes, pointing to a link with glucose-regulated K-ATP channels mediated by AMPK [14,16]. 

Molecular targets of AMPK in the β-cell, as well as their potential roles, have been documented and reviewed by others [3,4,5]. Several studies have reported that AMPK negatively regulates β-cell insulin exocytosis in primary rat islets and β-cell lines [14,18,19]. Pharmacological activation of AMPK in vitro by AICAR resulted in contradictory results, with either increased or decreased insulin secretion, alternatively having no effect on the secretory response [4]. In isolated mouse and rat islets, glucose-stimulated insulin secretion is inhibited after over-expression of constitutively active AMPK; however, introduction of a dominant negative form of AMPK has no effect on insulin release [20]. In the same study, transplantation of islets expressing the constitutively active AMPK into streptozotocin-induced diabetic mice improved glycemic control less effectively than transplantation with control islets. In contrast, other studies concluded that activation of AMPK by AICAR increases insulin secretion [4,5]. The effects of AMPK on glucose-stimulated insulin secretion may be influenced by experimental conditions, such as the glucose concentrations in cell culture media and during the insulin secretion assays. Chronic activation of the AMPKγ2 subunit in mice induces obesity and impairs insulin secretion [21]. While this phenotype is partially explained by ghrelin-induced hyperphagia, these findings call into question therapeutic strategies targeting AMPK. Thus, AMPK appears as a positive and negative regulator of insulin secretion. Current treatments for type 2 diabetes include metformin [22] and thiazolidinediones, which mediate some of their therapeutic effects by activation of AMPK [23], essentially through lowering hepatic glucose production. Troglitazone might provide β-cells “a rest” through activation of AMPK and inhibition of insulin hypersecretion when islets are exposed to high-glucose conditions [24]. Therefore, AMPK activation could play an important role by preserving β-cell integrity, while its action on the regulation of insulin secretion remains debated.

### 1.4. Effects of Metabolic Stresses on AMPK in the β-Cell 

Obesity and the pre-diabetic state are characterized by excessive nutrient supply from both the digestive tract and the internal stores, such as fatty acids from the adipose tissue and glucose from the liver. In vitro, chronic exposure of β-cells to metabolic stresses (e.g., glucose and fatty acids) impairs their function and potentially induces apoptosis [25]. In contrast to the acute potentiation of glucose-stimulated insulin secretion evoked by fatty acids, prolonged exposure induces β-cell lipo-dysfunction with elevated basal insulin release and impaired glucose response. The associated glucolipotoxicity concept, not demonstrated in patients [26], proposes that high glucose and fatty acids induce pleiotropic alterations associated with diabetes and the metabolic syndrome. In this context, metabolic stresses could lead to β-cell dysfunction and apoptosis [1,27]. However, little is known on AMPK responses to specific metabolic stressors, i.e., low versus high glucose, saturated versus unsaturated fatty acids, or chronic fructose exposure. Metabolic stresses, which may alter ATP production or accelerate its consumption, activate AMPK [28]. In pancreatic β-cells, AMPK is activated in conditions of nutrient deprivation (i.e., a fasting state) and accordingly should not be involved in the secretory response to a glucose rise. However, activated AMPK might prepare the β-cell for more robust secretion once stimulated by high glucose [17]. Here, we investigated the intracellular ATP signaling components (see Figure 1) in various metabolic stress conditions, i.e., glucose deprivation, glucotoxicity, lipotoxicity or prolonged treatment with fructose. The present work aimed at determining whether genes that encode either for AMPK subunits or for downstream targets and upstream kinases (defined as AMPK-associated genes) represent molecular targets of the main metabolic stresses in INS-1E β-cells and human islets.

## 2. Results

### 2.1. AMPK Profiling in β-Cells 

**Figure 2:**
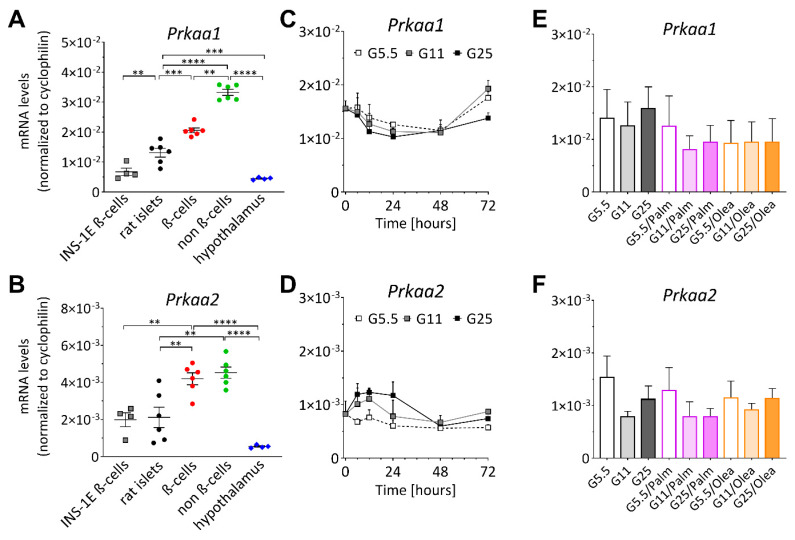
AMPK mRNA levels in rat islets and in INS-1E β-cells under metabolic stress conditions. (**A**–**B**) Relative expression of the two components of the AMPK catalytic subunits α1 and α2, encoded by the *Prkaa1* and *Prkaa2* genes, respectively; measured by qRT-PCR and normalized to cyclophilin (*n* = 6); ** *p*< 0.01, *** *p*< 0.005, **** *p*< 0.001. (**C**–**D**) Time course of the transcript levels in INS-1E β-cells after low (G5.5), control (G11) and high (G25) glucose exposure (*n* = 3). (**E**–**F**) Cells were treated for 3 days with 0.4 mM palmitate (Palm) or oleate (Olea) in the presence of 0.5% BSA (*n* = 5).

First, we analyzed the transcript levels of the two components of the AMPK catalytic subunits α1 and α2, encoded by the *Prkaa1* and *Prkaa2* genes, respectively, assuming it would reflect expression of the whole complex. Quantitative-RT-PCR analyses of *Prkaa1* and *Prkaa2* were performed in insulinoma cells and isolated rat islets, as well as FACS-purified rat β- and non β-cells. Both transcripts (*Prkaa1* and *Prkaa2*) were enriched in the whole rat islet and particularly abundant in sorted β-cells and non-β-cells compared to insulinoma INS-1E and cells isolated from the hypothalamus (Figure 2A,B). Of note, *Prkaa1* transcript levels were higher than those of *Prkaa2* in all fractions tested, with a relative difference of about 5-fold for the purified β-cells. 

At the protein level, there are limited data on the interaction of AMPK and other proteins/kinases. Moon and colleagues reported large-scale affinity purification–mass spectrometry analysis of the AMPK-α1 and -β1 subunits [29]. Numerous unique proteins (381) in the AMPKα/β interactome were identified and associated to β-cell functions when grouped into gene ontology terms. Those include the secretory response, cellular development, differentiation, cell–cell communication and actin organization, illustrating the broad range of functions mediated by AMPK activity.

### 2.2. Diabetogenic Conditions do not Alter AMPK Gene Expression in INS-1E β-Cells and Human Islets

The expression of the two AMPK catalytic subunits α1 and α2 genes was determined in INS-1E β-cells following chronic exposure to different metabolic stresses known to alter β-cell function. INS-1E β-cells are normally cultured at 11.1 mM glucose, which corresponds to their EC50 in terms of the secretory response, 5.5 and 25 mM corresponding, respectively, to the lower and upper plateau phases [30]. Cells were exposed for up to 3 days to 5.5 mM (G5.5, low) and 25 mM (G25, high) glucose. Chronic exposure of INS-1E β-cells to high glucose decreases glucose-stimulated insulin secretion and insulin content, alters differentiation via reduced expression of transcription factors and induces caspase 3 cleavage and cell death, revealing glucotoxicity [27,31]. In agreement with previous reports, chronic exposure to elevated concentrations of glucose drastically reduced expression of the β-cell transcription factors *Pdx1* and *Pparα* [27,32,33,34]. Time course studies revealed that AMPK mRNA levels (*Prkaa1* and *Prkaa2*) were not significantly altered over a period of 72h in INS-1E β-cells (Figure 2C,D). Palmitate and oleate were then added to the culture medium for 3 days, mimicking glucolipotoxic conditions (G25 with 0.4 mM palmitate or oleate). The expression profile of the two AMPK catalytic subunits, *Prkaa1* and *Prkaa2,* was not changed (Figure 2E,F), indicating that AMPK gene expression is not a target of the different tested metabolic stresses (i.e., glucose and fatty acids) in INS-1E β-cells. 

**Figure 3:**
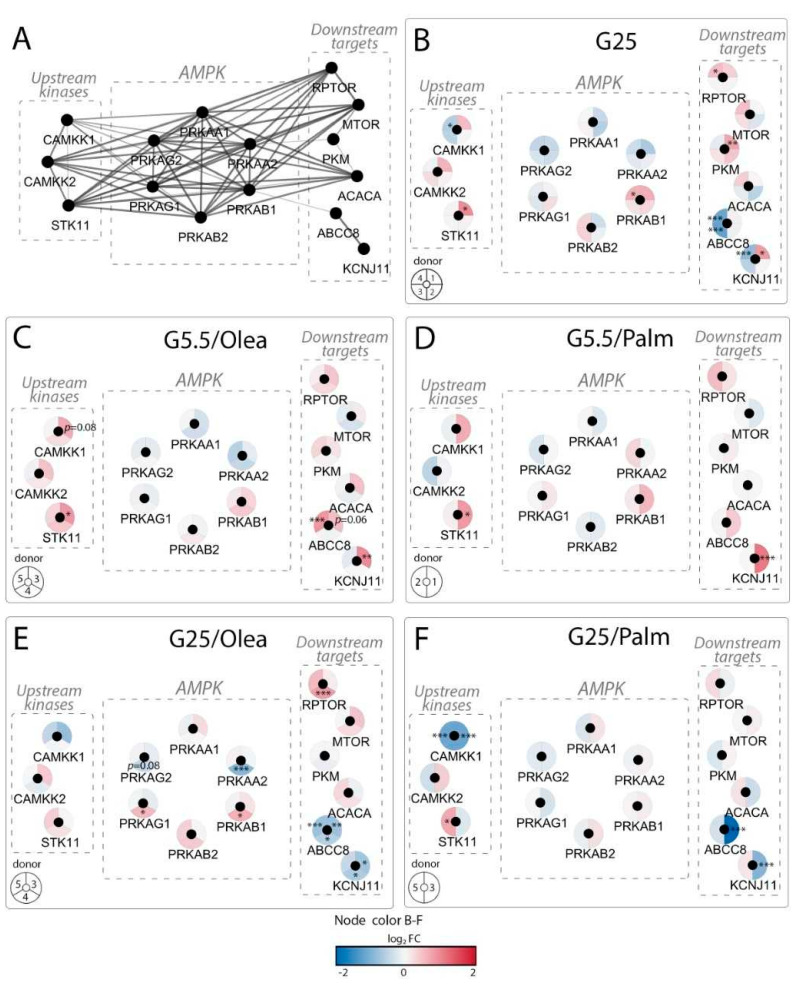
AMPK transcript levels in human islets under metabolic stress conditions. (**A**) Functional interaction network of human AMPK-associated genes, i.e., AMPK subunits (AMPK box), upstream kinases, and downstream targets. (**B**–**F**) Effects of culture conditions compared to standard G5.5 medium on transcript levels shown as up-regulated (red), down-regulated (blue), or unchanged (white). Each disk is split into individual changes for the different donors. (**B**) Genes regulated upon high-glucose conditions (G25). (**C–D**) Genes regulated upon (**C**) oleate or (**D**) palmitate exposure (0.4 mM) in control glucose condition (G5.5). (**E–F)** Genes regulated upon (**E**) oleate or (**F**) palmitate exposure (0.4 mM) in high-glucose conditions (G25). (**A**) Node connections were established according to the STRING interaction knowledgebase with a confidence score >0.4. Color code reflects the changes in expression in log2 fold changes (log2 FC; quantitative data in Supplementary Table S1) of that particular gene for each individual human donor (described in Table S2). Dashed boxes show, from left to right, the upstream kinases comprising *CAMKK* (calcium/calmodulin-dependent protein kinase kinase) and *STK11* (serine/threonine kinase 11/LKB1); the six subunits of AMPK; the downstream targets *RPTOR* (regulatory-associated protein of MTOR complex 1), *MTOR* (mechanistic target of rapamycin kinase), *PKM* (pyruvate kinase M1/2 isozyme), *ACACA* (acetyl-CoA carboxylase), *ABCC8* (SUR1 subunit), *KCNJ11* (KIR6.2 subunit). *adjusted *p* < 0.05, **adjusted *p* < 0.01, ***adjusted *p* < 0.001 between control 5.5 mM glucose and the specific culture condition. Clinical data of donors #1 to #5 are shown in Supplementary Table S2.

We also analyzed the expression profile of the different AMPK components in isolated human islets under the same metabolic stress conditions using RNA-Seq. Figure 3 presents a snapshot of the regulation of AMPK-associated genes from a whole-transcriptome data set (full data set not shown). We delineated a functional interaction network of AMPK-associated genes (Figure 3A) using the STRING knowledgebase [35,36] and represented the regulation of these genes at the transcript level under metabolic stressors (Figure 3B–F, Supplementary Table S1). All treatments were performed at 10% FCS to investigate the intrinsic effects of saturated versus unsaturated fatty acids without changing the standard culture conditions.

For clarity, the upstream kinases, the six subunits of AMPK and the downstream AMPK targets are delimited by dashed boxes (Figure 3A–F). Exposure of human islets to G25 for 3 days increased expression of the calcium/calmodulin-dependent protein kinase kinases *CAMKK1* and *CAMKK2*, as well as the liver serine/threonine kinase LKB1 (encoded by the *STK11* gene) (Figure 3B). G25 lowered *PRKAA1* and *PRKAA2* expression without changing the regulatory *PRKAB2*, *PRKAG1* and *PRKAG2* subunits. High glucose increased *PRKAB1* mRNA in one out of four donors (Supplementary Table S2). G25 did not modify the AMPK target genes, i.e., the mechanistic targets of rapamycin kinase *MTOR* and the key lipogenic enzyme acetyl-CoA carboxylase ACC (encoded by *ACACA*), while expression of the glycolytic enzyme pyruvate kinase M1/M2 isozyme (encoded by *PKM*) and the regulatory-associated protein of MTOR complex 1, *RPTOR*, was increased. Of note, the pyruvate kinase L/R isoform gene was not detected in human islets in any metabolic stress conditions. Interestingly, the expression of the K-ATP channel subunits KIR6.2 and SUR1, encoded respectively by *KCNJ11* and *ABCC8*, were significantly affected by glucotoxic conditions.

In human islets, neither oleate nor palmitate modified the six subunits of AMPK gene expression at physiological glucose G5.5 (Figure 3C,D). Treatment of islets with oleate at G5.5 for 3 days upregulated the kinase LKB1*/STK11* (Figure 3C) in one out of three donors. Palmitate also increased LKB1*/STK11* mRNA levels (Figure 3D), suggesting shared responses of the different fatty acids. Accordingly, addition of these fatty acids significantly increased the expression of the K-ATP channel subunits KIR6.2*/KCNJ11* and SUR1/*ABCC8*. Neither saturated nor unsaturated fatty acids modified expression of the AMPK target genes *PKM*, *MTOR*, *RPTOR* or *ACACA*. As shown in Figure 3E,F, at high glucose (G25) oleate and palmitate decreased expression of *CAMKK1*, potentially impairing AMPK activation, as well as the expression of the K-ATP channel subunits KIR6.2/*KCNJ11* and SUR1/*ABCC8*. Similar to G25 alone (Figure 3B), oleate at high glucose lowered *PRKAA2* expression and increased *PRKAB1* mRNA in one out of three donors. *PRKAG1* and *RPTOR* were specifically upregulated by oleate compared to G25 alone. Expression level of AMPK subunits was not altered by G25 and palmitate. Taken together, the expression profile of the six subunits of AMPK was not profoundly modified by the different diabetogenic conditions in human islets, exhibiting marginal stress-specific signatures. However, gene expression of the upstream kinases as well as the downstream AMPK targets was significantly affected by glucotoxic and glucolipotoxic conditions. In particular, concomitant downregulation of *CAMKK1* and K-ATP channel subunits may alter metabolism–secretion coupling upon fatty acid exposure.

### 2.3. Glucotoxic Conditions and Chronic Fructose Exposure do not Alter AMPK Protein Levels in INS-1E β-Cells and Human Islets, While Prolonged Treatment with Fructose Activates AMPK

**Figure 4:**
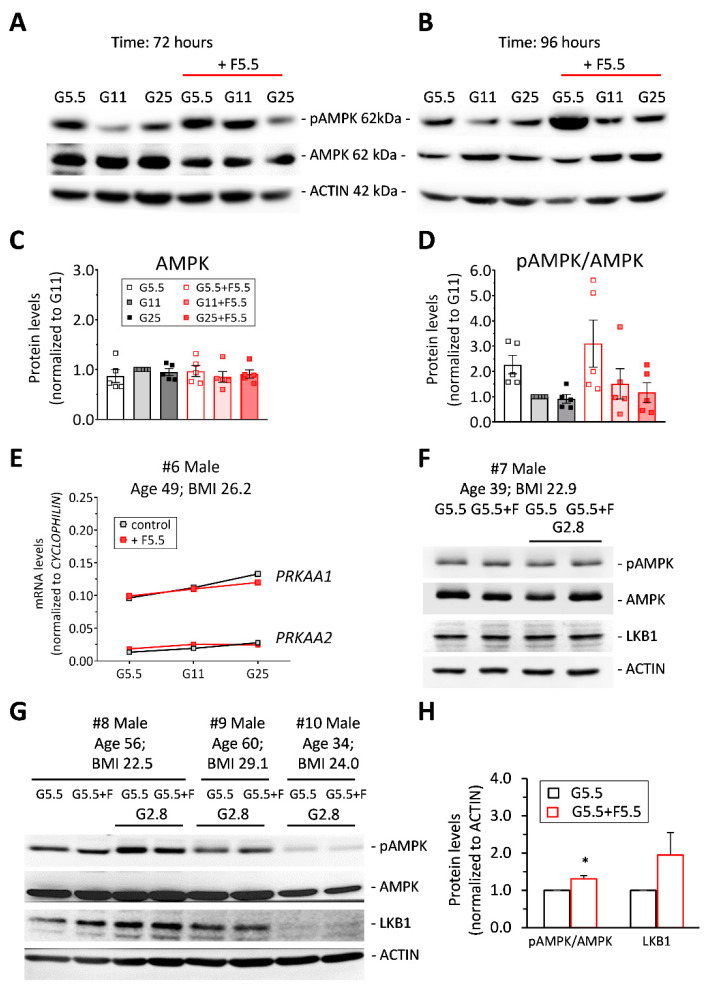
AMPK activation in INS-1E β-cells and in human islets exposed to high glucose and fructose. (**A–D**) INS-1E β-cells were cultured for (**A**) 3 days or for (**B**) 4 days in low (G5.5), standard (G11) and high (G25) glucose supplemented with 5.5 mM fructose (red line). Representative immunoblotting showing levels of pAMPKα, AMPKα (AMPK) and ACTIN in cells right after the culture period. Quantitative analysis of (**C**) AMPK and (**D**) pAMPK/AMPK band densities normalized to ACTIN are shown for 3-day-treated cells (*n* = 5). Results are expressed as protein levels normalized to G11 values. (**E-H**) Freshly isolated human islets from six donors were treated for 4 days with 5.5 mM fructose (F) in complete CRML-1066 medium. Culture at 5.5 mM glucose (G5.5) served as a control culture condition. (**E**) Transcript levels of AMPK catalytic subunits α1 (*PRKAA1*) and α2 (*PRKAA2*) were quantified in a batch of islets from one representative human islet preparation exposed to different glucose concentrations, normalized to cyclophilin. (**F-G**) Representative immunoblotting showing levels of pAMPK, AMPK, LKB1 and ACTIN from treated islets isolated from four donors right after culture and after 1 h post-culture of glucose starving at 2.8 mM (G2.8). (**H**) Quantitative analysis of pAMPK/AMPK and LKB1 band densities normalized to ACTIN is shown (*n* = 6). Results are expressed as protein levels normalized to G5.5 control values. *p < 0.05 G5.5+F islets versus G5.5 islets.

We then further investigated the apparently paradoxical effects of the two monosaccharides, glucose and fructose, on AMPK activation. INS-1E β-cells were chronically treated over days with different glucose concentrations plus 5.5 mM fructose (F5.5). Cultures in standard RPMI-1640 medium served as control (G11). Western blot analysis showed that chronic exposure to fructose at low glucose (G5.5) induced AMPKα phosphorylation (1.4-fold, *p* < 0.05) compared to naïve INS-1E β-cells (Figure 4A,B,D). Compared to the low glucose G5.5 culture condition, higher glucose concentrations in the culture medium reduced AMPK activation by 57% (at G11) and 69% (at G25) in control cells and by 51% (at G11) and 62% (at G25) in cells treated with both fructose and glucose. This indicates that AMPK response per se to glucose was preserved in the presence of fructose. Culturing cells at low (G5.5) and high (G25) glucose concentrations in the presence or absence of fructose during 3 days did not modify AMPKα (detected as α1 and α2 catalytic subunits) protein levels (Figure 4C). Of note, quantitative-RT-PCR analysis for *Prkaa1* and *Prkaa2* performed in parallel with immunoblotting experiments confirmed that AMPK mRNA levels remained unchanged in INS-1E β-cells exposed to glucose (Figure 2E,F) and/or fructose (5.5 mM) for 3-4 days (not shown). 

Finally, we investigated the effects of prolonged treatment with fructose on AMPK activation in human islets exposed for 4 days to 5.5 mM fructose (F5.5) in standard culture medium containing 5.5 mM glucose (G5.5, used for control groups). Similar to INS-1E β-cells, AMPK catalytic subunits α1 (*PRKAA1)* and α2 (*PRKAA2*) were expressed in human islets, *PRKAA1* transcripts being more abundant compared with *PRKAA2* (Figure 4E). This pattern was changed neither by the different glucose concentrations nor by the presence of fructose in the culture medium (Figure 4E). Chronic exposure to fructose did not modify AMPK protein levels compared to control islets, either at the end of the culture period or following an additional 1 h starving period at 2.8 mM glucose (G2.8) (Figure 4F,G). Of note, the G2.8 incubation caused a relative increase of AMPK phosphorylation in both control and fructose-treated islets, indicating that the acute AMPK response to glucose was preserved following chronic fructose treatment, while remaining higher than in control cells. After 4 days of fructose treatment, AMPK phosphorylation levels were increased 1.3-fold (*p* < 0.05, Figure 4H) on average in six human islets of different donors (Supplementary Table S2, donors #11 and #12 are not shown). There was a trend for increased protein levels of the upstream AMPK-regulating kinase LKB1 compared with control human islets (Figure 4F–H). Thus, chronic exposure of human islets to fructose increased AMPK phosphorylation levels, an effect associated with the potentiation of glucose-stimulated insulin secretion [12]. 

Overall, fructose treatment induced intracellular fasting-like phenotype in INS-1E β-cells and human islets, uncovered by AMPK activation. Previously published data indicate that fructose activates extracellular ATP signaling (see Figure 1), an effect mediated by the activation of purinergic P2Y1 receptors through increased release of cellular ATP through Panx1 channels [12]. Activation of extracellular ATP signaling enhances insulin exocytosis. These cellular mechanisms underscore the potentiating effects of fructose on the β-cell response at intermediate physiological glucose concentrations, raising insulin secretion to non-physiological levels that might favor obesity.

## 3. Discussion

In the context of obesity and diabetes, AMPK is of particular interest since it functions as a central mediator of the cellular response to metabolic stress and mitochondrial insults, being activated under conditions of energy deficiency [13]. Thus, it plays a central role as a regulator of energy metabolism, coordinating catabolism and anabolism [37]. Exercise also activates skeletal muscle AMPK, as reviewed elsewhere [38]. Skeletal muscle AMPK regulates fatty acid metabolism, exercise capacity, mitochondrial biogenesis and contraction-stimulated glucose uptake. AMPK has been shown to upregulate glucose transporter and fatty acid translocase in cardiomyocytes and to inhibit the synthesis of fatty acids and cholesterol in hepatocytes. In the context of diabetes, one of the most salient actions of AMPK, once activated, is found in the liver, i.e., the inhibition of gluconeogenesis, which participates in hyperglycemia. Thus, AMPK is considered to be a central mediator in the management of type 2 diabetes, in particular for the numerous patients treated with metformin, which activates AMPK [22]. 

In obese patients, the pre-diabetic state is accompanied by hyperinsulinemia as a consequence of a primary resistance of peripheral tissues to the action of insulin. In case of significant β-cell loss, such a state may evolve towards hyperglycemia and diabetes [2]. Interestingly, an early limitation of the full secretory capacity of the β-cell before the obese state prevents diet-induced obesity and hyperinsulinemia in mice. This was demonstrated by both the reduction in insulin gene dosage [39] and the abrogation of the amplifying pathway through the knockout of glutamate dehydrogenase [40]. The pharmacological inhibition of glutamate dehydrogenase can be achieved by using epigallocatechin-3-gallate (EGCG), which lowers glucose-stimulated insulin secretion in human pancreatic islets [41] and reduces the body weight of obese subjects [42]. Similar effects have been reported in various animal models [43,44], including improvement of glucose tolerance in diabetic db/db mice [45]. Interestingly, EGCG also activates AMPK in human islets and C2C12 myotubes along with increased glucose uptake in human myotubes [41]. These combined effects might have a protective role in the pre-diabetic state.

Fructose used as a dietary sweetener is associated with obesity and diabetes. This monosaccharide was introduced in processed food as a taste enhancer in the form of high-fructose corn syrup (HFCS) in the late 70s, [46,47]. The cellular mechanisms remain obscure, while data accumulates for a causal link between dietary fructose and metabolic dysfunctions. In contrast to glucose, humans are not physiologically equipped for rapid metabolism of a large fructose load, such as heavy consumption of HFCS-enriched beverages. It is thought that most of the circulating fructose is taken up by tissues expressing the sugar transporters GLUT5 and GLUT2 [48]. As opposed to the ubiquitous energy substrate glucose, fructose catabolism is essentially restricted to the liver and not limited by metabolic feedback. Thus, a high-fructose diet increases de-novo fatty acid synthesis and triglyceride formation, favoring hepatic steatosis. Regarding the apparent paradox of a sugar-induced depletion of cellular ATP, it is striking to note that central fructose lowers hypothalamic ATP levels as opposed to glucose. Accordingly, prolonged central administration of fructose increases AMPK phosphorylation in the hypothalamus of treated rats [49], an effect also observed in cultured hypothalamic GT1-7 cells [50] and associated with higher food intake [51]. Thus, in the brain, fructose alters cellular energy sensing mediated by AMPK activation. Given the close relationship between these two glucose sensors, i.e., the hypothalamus and the β-cell [52], one can hypothesize that similar mechanisms govern the response of β-cells to chronic exposure to fructose, favoring the reported weight gain associated with this sugar consumption. We recently reported that chronic fructose exposure on pancreatic β-cells results in AMPK activation and exaggerated insulin secretion, even at intermediate glucose concentrations [12]. Overall, fructose induces an intracellular ATP signaling axis, potentiating glucose-stimulated insulin secretion. Fructose reduces intracellular ATP levels, without affecting mitochondrial respiration, and activates AMPK (Figure 1 and [12]). Regarding extracellular signaling, fructose activates Panx1 channels promoting the release of cellular ATP and auto/paracrine activation of purinergic P2Y1 receptors, thereby enhancing the secretory response.

## 4. Materials and Methods

### 4.1. Reagents

D-glucose, fatty acids, D-fructose, culture media, and other basic reagents were obtained from Sigma-Aldrich (St.-Louis, MO, USA).

### 4.2. Cell Culture, Human Islets and Treatments

Rat INS-1E β-cells [30] were grown in RPMI-1640 medium at 11.1 mM glucose supplemented with 5% (vol./vol.) heat-inactivated fetal calf serum (FCS). At day 4 day after seeding, cells were further cultured for 3 days at either 11.1 mM glucose (G11, control) or exposed to low 5.5 mM (G5.5) and high 25 mM (G25) glucose concentrations. INS-1E β-cells were also exposed to 0.4 mM palmitate (Palm; saturated fatty acid C16:0) or 0.4 mM oleate (Olea; unsaturated fatty acid C18:1) in the presence of 0.5% BSA, as detailed previously [27,33]. Stock solutions of fatty acids bound to BSA were adjusted to 10 mM fatty acids using 1.8-mM fatty-acid-free BSA and were stored at −20 °C under nitrogen [53]. The different treatments were systematically performed in parallel cultures. For fructose exposure experiments, cells were maintained after seeding for 3–4 days at low (G5.5), control (G11) and high (G25) glucose supplemented with 5.5 mM fructose (+F5.5) as described [12]. 

Human islets were isolated at the Hôpitaux Universitaires de Genève (Switzerland) from pancreata of deceased organ donors (*n* = 14) who had provided written informed consent (ECIT consortium, http://ecit.dri-sanraffaele.org/, accessed on 01/06/2020). Donors had an average BMI of 25.7 ± 2.6 kg/m^2^ and were aged 50.7 ± 8.1 years. None of the donors were diagnosed with metabolic syndrome or diabetes (see clinical data in Supplementary Table S2). Islets were maintained for standard recovery period of time (1–3 days) in CMRL-1066 medium at 5.5 mM glucose supplemented with 10% FCS and used for experiments straight away without shipping maneuver (isolation and experiment being performed in the same institution). Then, isolated islets were hand-picked, washed and further exposed for 3 days, in the presence of 10% FCS, to high glucose G25, to oleate (0.4 mM) at G5.5, to palmitate (0.4 mM) at G5.5, to oleate (0.4 mM) at G25, and to palmitate (0.4 mM) at G25, while the physiological 5.5 mM glucose served as control [27,54]. 

### 4.3. RNA-Seq

RNA was isolated from cultured human islets following standard procedures [54]. RNA sequencing methodology was described elsewhere [55]. Briefly, RNA was fragmented and cDNA synthesis was performed according to the manufacturer’s instructions (TruSeq 2, Illumina Inc, San Diego, CA, USA). Mapped reads were analyzed by R/Bioconductor package EdgeR v. 3.4.2, for differential expression analysis. The counts were normalized according to the library size and filtered. Transcript abundances were compared in pairwise conditions in a modified Fischer’s exact test (as implemented in edgeR). An EdgeR procedure was used to analyze count data for significance without replicates and for estimating the dispersion. The differentially expressed genes’ *p*-values were corrected for multiple testing error with an adjusted *p* value < 0.05. The correction used was Benjamini–Hochberg (BH). We built functional networks of AMPK-associated genes using Cytoscape StringApp, including both physical interactions from experimental data and functional associations from curated pathways, automatic text mining, and prediction methods, with a confidence score of 0.4 [35]. 

### 4.4. Animals and Islet Isolation

Rat handling from in-house breeding was carried out in our local certified animal facility (CMU-zootechnie, Geneva, Switzerland) according to procedures that were approved by the animal care and experimentation authorities of the Canton of Geneva. Pancreatic islets of Langerhans were isolated from male rats (200–250 g, 7–8 weeks old) by collagenase digestion as described elsewhere [56]. The yield was about 600–800 islets per rat. Immediately after isolation, islets were hand-picked, washed twice, trypsinized and islet cells then sorted. Enriched fractions of β-cells were separated from α-cells and exocrine cells by autofluorescence-activated sorting using a FACS Vantage SE cell sorter (Becton Dickinson, Franklin Lakes, N.J., USA) following 2 criteria: FAD auto-fluorescence intensity (excitation wavelength at 488 nm) and the cell size, as described previously [56].

### 4.5. Isolation of RNA and Quantitative RT-PCR

INS-1E β-cells were cultured in 6-well dishes as described above. Total RNA was extracted with the Trizol reagent (Invitrogen, Carlsbad, CA, USA) and 2 µg were converted into cDNA as described previously [12]. Primers for *Prkaa1*, *Prkaa2* and *cyclophilin* (*PPia*) were designed using the Primer Express Software (Applera Europe, Rotkreutz, Switzerland); see list of primers in Supplementary Table S3. QT-RT-PCR was performed on insulinoma cells and isolated rat islets, as well as FACS-purified β- and α-cells using an ABI 7000 Sequence Detection System (Applera, Norwalk, CT, USA). PCR products were quantified fluorometrically using the SYBR Green Master kit (Roche, Mannheim, Germany). Two distinct amplifications, derived from at least six independent experiments, were performed in duplicate for each transcript, and mean values were normalized to those of the reference mRNA cyclophilin.

### 4.6. Immunoblotting

At the end of the culture periods, protein extracts from total INS-1E β-cells (4.0 × 10^6^ cells/dish) and human islets (200 islets/well) were harvested in lysis buffer as described [33,54]. Proteins from total cell extracts (10–20 μg/lane) were separated by 8%–10% SDS-PAGE before transfer onto nitrocellulose membrane. The membrane was then probed overnight at 4 °C with rabbit polyclonal antibodies against AMPKα (62 kDa, the antibody detects both the α1 and α2 isoforms of the catalytic subunit, not the regulatory β or γ subunits); p-AMPKα (thr172) (1:1000 dilutions, Cell Signaling Technology, Danvers, MA, USA); mouse monoclonal antibodies against LKB1 (1:200, Santa Cruz Biotechnology, Santa Cruz, CA, USA); and ACTIN (1:5000, Chemicon-Millipore, Zug, Switzerland). After washing, the membranes were incubated for 1 h at RT with secondary horseradish peroxidase-conjugated anti-rabbit or anti-mouse IgG antibodies (1:10000, Amersham Biosciences, Buckinghamshire, UK) according to primary antibodies. Proteins were revealed by chemiluminescence (ECL, Amersham Biosciences, Buckinghamshire, UK), analyzed with the ChemiDoc XRS System (Bio-Rad, Hercules, CA, USA), and bands were quantified with Scion Image software (Scion Corporation, Frederick, MD, USA).

### 4.7. Statistical Analysis

Results are presented as means ± SEM. Statistical tests between values of treated and control cells were performed using one-way ANOVA analysis followed by least significant difference post-hoc tests when multiple comparisons were made. Statistical analyses were performed using GraphPad Prism 7 software. Where appropriate, a two-tailed paired t-test was performed. A *p*-value lower than 0.05 was considered statistically significant.

## 5. Conclusions

In the context of diabetes, various conditions have been proposed to trigger damage to insulin-secreting cells. Among them, chronic high glucose, fatty acids, and oxidative attacks are currently highlighted as the main stressors. However, the respective contributions of different diabetes-associated metabolic stresses to the dysfunction of β-cells remains unclear. Here, we explored the AMPK responses to different metabolic stresses at mRNA and protein levels side-by-side in INS-1E β-cells and human islets. Bioinformatics analysis showed that expression profiles of the six subunits of AMPK genes are modestly modified by the different diabetogenic conditions, exhibiting limited nutrient stress-specific signatures. The β-cells adapted their AMPK responses to metabolic stresses mainly by phosphorylation/dephosphorylation, preserving the corresponding expression profile. However, expression of AMPK upstream kinases LKB1 and CAMKK, as well as downstream targets KIR6.2 and SUR1, was significantly modified by glucotoxic and glucolipotoxic conditions.

The contribution of AMPK in the control of glucose homeostasis may appear controversial. Indeed, AMPK is activated as a sign of cellular energy scarcity, which is counteracted by the provision of glucose. However, in the liver, AMPK activation inhibits gluconeogenesis and, as a consequence, stops hepatic glucose production, leaving glucose-dependent organs at risk of energy deprivation. In the β-cells, AMPK is activated concomitantly with the arrest of insulin secretion when glycemia goes down, while some studies have reported beneficial effects of AMPK activation for β-cell function. These apparent paradoxes illustrate the possible conflicts between survival of specific cells and benefits for the whole organism. AMPK activation might represent a survival mechanism, in particular for β-cells in a pre-diabetic state.

## Supplementary Materials

Supplementary materials can be found at https://www.mdpi.com/1422-0067/21/11/3982/s1.

## References

[1] Prentki M., Peyot M.L., Masiello P., Madiraju S.R.M. (2020). Nutrient-Induced Metabolic Stress, Adaptation, Detoxification, and Toxicity in the Pancreatic β-Cell. Diabetes.

[2] Kasuga M. (2006). Insulin resistance and pancreatic beta cell failure. J. Clin. Investig..

[3] Rourke J.L., Hu Q., Screaton R.A. (2018). AMPK and Friends: Central Regulators of β Cell Biology. Trends Endocrinol. Metab..

[4] Fu A., Eberhard C.E., Screaton R.A. (2013). Role of AMPK in pancreatic β cell function. Mol. Cell. Endocrinol..

[5] Szkudelski T., Szkudelska K. (2019). The relevance of AMP-activated protein kinase in insulin-secreting β cells: A potential target for improving β cell function?. J. Physiol. Biochem..

[6] Maechler P. (2013). Mitochondrial function and insulin secretion. Mol. Cell. Endocrinol..

[7] Maechler P. (2017). Glutamate pathways of the β-cell and the control of insulin secretion. Diabetes Res. Clin. Pract..

[8] Prentki M., Matschinsky F.M., Madiraju S.R. (2013). Metabolic signaling in fuel-induced insulin secretion. Cell Metab..

[9] Henquin J.C. (2012). Do pancreatic β cells “taste” nutrients to secrete insulin?. Sci. Signal..

[10] Malaisse W.J. (2014). Insulin release: The receptor hypothesis. Diabetologia.

[11] Wollheim C.B., Maechler P. (2015). β cell glutamate receptor antagonists: Novel oral antidiabetic drugs?. Nat Med..

[12] Bartley C., Brun T., Oberhauser L., Grimaldi M., Molica F., Kwak B.R., Bosco D., Chanson M., Maechler O. (2019). Chronic fructose renders pancreatic β-cells hyper-responsive to glucose-stimulated insulin secretion through extracellular ATP signaling. Am. J. Physiol. Endocrinol. Metab..

[13] Herzig S., Shaw R.J. (2018). AMPK: Guardian of metabolism and mitochondrial homeostasis. Nat. Rev. Mol. Cell Biol..

[14] Salt I.P., Johnson G., Ashcroft S.J., Hardie D.G. (1998). AMP-activated protein kinase is activated by low glucose in cell lines derived from pancreatic β cells, and may regulate insulin release. Biochem. J..

[15] da Silva Xavier G., Leclerc I., Salt I., Doiron B., Hardie D.G., Kahn A., Rutter G.A. (2000). Role of AMP-activated protein kinase in the regulation by glucose of islet β cell gene expression. Proc. Natl. Acad. Sci. USA.

[16] Smith A.J., Partridge C.J., Asipu A., Mair L.A., Hunter M., Sivaprasadarao A. (2006). Increased ATP-sensitive K+ channel expression during acute glucose deprivation. Biochem. Biophys. Res. Commun..

[17] Lim A., Park S.H., Sohn J.W., Jeon J.H., Park J.H., Song D.K., Lee S.H., Ho W.K. (2009). Glucose deprivation regulates KATP channel trafficking via AMP-activated protein kinase in pancreatic β-cells. Diabetes.

[18] da Silva Xavier G., Leclerc I., Varadi A., Tsuboi T., Moule S.K., Rutter G.A. (2003). Role for AMP-activated protein kinase in glucose-stimulated insulin secretion and preproinsulin gene expression. Biochem. J..

[19] Rutter G.A., Da Silva Xavier G., Leclerc I. (2003). Roles of 5’-AMP-activated protein kinase (AMPK) in mammalian glucose homoeostasis. Biochem. J..

[20] Richards S.K., Parton L.E., Leclerc I., Rutter G.A., Smith R.M. (2005). Over-expression of AMP-activated protein kinase impairs pancreatic β-cell function in vivo. J. Endocrinol..

[21] Yavari A., Stocker C.J., Ghaffari S., Wargent E.T., Steeples V., Czibik G., Pinter K., Bellahcene M., Woods A., de Morentin P.B.M. (2016). Chronic Activation of γ2 AMPK Induces Obesity and Reduces β Cell Function. Cell Metab..

[22] Rena G., Hardie D.G., Pearson E.R. (2017). The mechanisms of action of metformin. Diabetologia.

[23] Fryer L.G., Parbu-Patel A., Carling D. (2002). The Anti-diabetic drugs rosiglitazone and metformin stimulate AMP-activated protein kinase through distinct signaling pathways. J. Biol. Chem..

[24] Deng R., Nie A., Jian F., Liu Y., Tang H., Zhang J., Zhang Y., Shao L., Li F., Zhou L. (2014). Acute exposure of β-cells to troglitazone decreases insulin hypersecretion via activating AMPK. Biochim. Biophys. Acta.

[25] Bensellam M., Laybutt D.R., Jonas J.C. (2012). The molecular mechanisms of pancreatic β-cell glucotoxicity: Recent findings and future research directions. Mol. Cell. Endocrinol..

[26] Weir G.C. (2020). Glucolipotoxicity, β-Cells, and Diabetes: The Emperor Has No Clothes. Diabetes.

[27] Oberhauser L., Granziera S., Colom A., Goujon A., Lavallard V., Matile S., Roux A., Brun T., Maechler P. (2020). Palmitate and oleate modify membrane fluidity and kinase activities of INS-1E β-cells alongside altered metabolism-secretion coupling. Biochim. Biophys. Acta Mol. Cell Res..

[28] Hardie D.G., Ross F.A., Hawley S.A. (2012). AMPK: A nutrient and energy sensor that maintains energy homeostasis. Nat. Rev. Mol. Cell Biol..

[29] Moon S., Han D., Kim Y., Jin J., Ho W.K., Kim Y. (2014). Interactome analysis of AMP-activated protein kinase (AMPK)-α1 and -β1 in INS-1 pancreatic β-cells by affinity purification-mass spectrometry. Sci. Rep..

[30] Merglen A., Theander S., Rubi B., Chaffard G., Wollheim C.B., Maechler P. (2004). Glucose sensitivity and metabolism-secretion coupling studied during two-year continuous culture in INS-1E insulinoma cells. Endocrinology.

[31] Roche E., Farfari S., Witters L.A., Assimacopoulos-Jeannet F., Thumelin S., Brun T., Corkey B., Saha A., Prentki M. (1998). Long-term exposure of β-INS cells to high glucose concentrations increases anaplerosis, lipogenesis, and lipogenic gene expression. Diabetes.

[32] Brun T., Maechler P. (2016). β-cell mitochondrial carriers and the diabetogenic stress response. Biochim. Biophys. Acta.

[33] Brun T., Scarcia P., Li N., Gaudet P., Duhamel D., Palmieri F., Maechler P. (2013). Changes in mitochondrial carriers exhibit stress-specific signatures in INS-1Eβ-cells exposed to glucose versus fatty acids. PLoS ONE.

[34] Ravnskjaer K., Boergesen M., Dalgaard L.T., Mandrup S. (2006). Glucose-induced repression of PPARα gene expression in pancreatic β-cells involves PP2A activation and AMPK inactivation. J. Mol. Endocrinol..

[35] Doncheva N.T., Morris J.H., Gorodkin J., Jensen L.J. (2019). Cytoscape StringApp: Network Analysis and Visualization of Proteomics Data. J. Proteome Res..

[36] Szklarczyk D., Gable A.L., Lyon D., Junge A., Wyder S., Huerta-Cepas J., Simonovic M., Doncheva N.T., Morris J.H., Bork P. (2019). STRING v11: Protein-protein association networks with increased coverage, supporting functional discovery in genome-wide experimental datasets. Nucleic Acids Res..

[37] Hardie D.G., Scott J.W., Pan D.A., Hudson E.R. (2003). Management of cellular energy by the AMP-activated protein kinase system. FEBS Lett..

[38] O’Neill H.M., Holloway G.P., Steinberg G.R. (2013). AMPK regulation of fatty acid metabolism and mitochondrial biogenesis: Implications for obesity. Mol. Cell. Endocrinol..

[39] Mehran A.E., Templeman N.M., Brigidi G.S., Lim G.E., Chu K.-Y., Hu X., Botezelli J.D., Asadi A., Hoffman B., Kieffer T.J. (2012). Hyperinsulinemia drives diet-induced obesity independently of brain insulin production. Cell Metab..

[40] Vetterli L., Carobbio S., Frigerio F., Karaca M., Maechler P. (2016). The Amplifying Pathway of the β-Cell Contributes to Diet-induced Obesity. J. Biol. Chem..

[41] Pournourmohammadi S., Grimaldi M., Stridh M.H., Lavallard V., Waagepetersen H.S., Wollheim C.B., Maechler P. (2017). Epigallocatechin-3-gallate (EGCG) activates AMPK through the inhibition of glutamate dehydrogenase in muscle and pancreatic ss-cells: A potential beneficial effect in the pre-diabetic state?. Int. J. Biochem. Cell Biol..

[42] Basu A., Sanchez K., Leyva M.J., Wu M., Betts N.M., Aston C.E., Lyons T.J. (2010). Green tea supplementation affects body weight, lipids, and lipid peroxidation in obese subjects with metabolic syndrome. J. Am. Coll. Nutr..

[43] Sae-tan S., Grove K.A., Lambert J.D. (2011). Weight control and prevention of metabolic syndrome by green tea. Pharmacol. Res..

[44] Wang S., Moustaid-Moussa N., Chen L., Mo H., Shastri A., Su R., Bapat P., Kwun I., Shen C.-L. (2014). Novel insights of dietary polyphenols and obesity. J. Nutr. Biochem..

[45] Ortsater H., Grankvist N., Wolfram S., Kuehn N., Sjoholm A. (2012). Diet supplementation with green tea extract epigallocatechin gallate prevents progression to glucose intolerance in db/db mice. Nutr. Metab. (Lond.).

[46] Bray G.A., Nielsen S.J., Popkin B.M. (2004). Consumption of high-fructose corn syrup in beverages may play a role in the epidemic of obesity. Am. J. Clin. Nutr..

[47] Johnson R.J., Segal M.S., Sautin Y., Nakagawa T., Feig D., Kang D.-H., Gersch M.S., Benner S., Sanchez-Lozada L.G. (2007). Potential role of sugar (fructose) in the epidemic of hypertension, obesity and the metabolic syndrome, diabetes, kidney disease, and cardiovascular disease. Am. J. Clin. Nutr..

[48] Tappy L., Le K.A. (2010). Metabolic effects of fructose and the worldwide increase in obesity. Physiol. Rev..

[49] Kinote A., A Faria J., A Roman E., Solon C., Razolli D.S., Ignacio-Souza L., Sollon C.S., Nascimento L.F., De Araújo T.M., Barbosa A.P.L. (2012). Fructose-induced hypothalamic AMPK activation stimulates hepatic PEPCK and gluconeogenesis due to increased corticosterone levels. Endocrinology.

[50] Burmeister M.A., Ayala J., Drucker D.J., Ayala J.E. (2013). Central glucagon-like peptide 1 receptor-induced anorexia requires glucose metabolism-mediated suppression of AMPK and is impaired by central fructose. Am. J. Physiol. Endocrinol. Metab..

[51] Cha S.H., Wolfgang M., Tokutake Y., Chohnan S., Lane M.D. (2008). Differential effects of central fructose and glucose on hypothalamic malonyl-CoA and food intake. Proc. Natl. Acad. Sci. USA.

[52] Yang X.J., Kow L.M., Funabashi T., Mobbs C.V. (1999). Hypothalamic glucose sensor: Similarities to and differences from pancreatic β-cell mechanisms. Diabetes.

[53] Frigerio F., Brun T., Bartley C., Usardi A., Bosco D., Ravnskjær K., Mandrup S., Maechler P. (2010). Peroxisome proliferator-activated receptor alpha (PPARα) protects against oleate-induced INS-1E β cell dysfunction by preserving carbohydrate metabolism. Diabetologia.

[54] Brun T., Li N., Jourdain A.A., Gaudet P., Duhamel D., Meyer J., Bosco D., Maechler P. (2015). Diabetogenic milieus induce specific changes in mitochondrial transcriptome and differentiation of human pancreatic islets. Hum. Mol. Genet..

[55] Schmidt S.F., Madsen J.G., Frafjord K.O., Poulsen L., Salo S., Boergesen M., Loft A., Larsen B.D., Madsen M.S., Holst J.J. (2016). Integrative Genomics Outlines a Biphasic Glucose Response and a ChREBP-RORγ Axis Regulating Proliferation in β Cells. Cell Rep..

[56] Rouiller D.G., Cirulli V., Halban P.A. (1991). Uvomorulin mediates calcium-dependent aggregation of islet cells, whereas calcium-independent cell adhesion molecules distinguish between islet cell types. Dev. Biol..

