# Supplementary material for: AMPK Profiling in Rodent and Human Pancreatic Beta-Cells under Nutrient-Rich Metabolic Stress
Source: Int J Mol Sci. 2020 Jun 1;21(11):3982. doi: 10.3390/ijms21113982 (PMC7312098; doi:10.3390/ijms21113982)
Supplement: Supplementary file 1 [file ijms-21-03982-s001.pdf]

Brun *et al*. **Supplementary files**

AMPK profiling in rodent and human pancreatic beta-cells under nutrient-rich metabolic stress

Supplemental Table S1: Quantitative data related to the transcriptomic profiles of AMPK-associated genes of human islets under different metabolic stresses.

Supplementary Table S2: Clinical Data related to human islets

Supplementary Table S3: Rat and human primers used for quantitative RT-PCR analysis

Supplemental information Table S1: Quantitative data related to the transcriptomic profiles of AMPK-associated genes of human islets under different metabolic stresses.

		Donor #1		Donor #2		Donor #3		Donor #4		Donor #5	
		log2 FC	p adj	log2 FC	p adj	log2 FC	p adj	log2 FC	p adj	log2 FC	p adj
G25	PRKAA1	-0.42831342	0.16654696	-0.56726992	0.12152884	0.0567878	0.95372166	0.01620298	0.98271495	NA	NA
	PRKAA2	-0.66494688	0.12029618	-0.13017463	0.93477811	-0.21777626	0.69120286	-0.39350653	0.18098591	NA	NA
	PRKAB1	0.6762521	0.07096901	0.30198245	0.55904086	0.20929898	0.67662011	0.6520113	0.01363645	NA	NA
	PRKAB2	-0.36960339	0.32701834	-0.0937707	0.92395808	0.34312064	0.4128375	0.32944334	0.26102151	NA	NA
	PRKAG1	-0.05927429	0.94773709	0.2429154	0.60119097	-0.24960104	0.59289296	-0.17436253	0.5910228	NA	NA
	PRKAG2	-0.44267163	0.23543569	-0.40415471	0.35794498	-0.27283055	0.56600041	-0.4926408	0.06478275	NA	NA
	STK11	0.98217715	0.01077677	0.06999019	0.96296821	0.03007558	0.99055702	0.05952606	0.88118264	NA	NA
	CAMKK1	0.55135249	0.20379345	-0.03548186	1	-0.53833293	0.16899806	-0.58021163	0.03016005	NA	NA
	CAMKK2	0.62244535	0.09980416	0.06717643	0.96803951	0.2650393	0.59017252	-0.07261492	0.86713887	NA	NA
	ACACA	0.02169252	1	-0.52746313	0.14249328	-0.17643036	0.77398209	0.38301186	0.16929891	NA	NA
	MTOR	0.0103755	1	-0.26929589	0.56683119	0.24928203	0.62138171	0.51712911	0.05493795	NA	NA
	RPTOR	0.35668739	0.44863895	0.03768111	0.98392317	0.0208219	1	0.53641652	0.04972474	NA	NA
	ABCC8	0.07063173	0.86392464	-0.08596365	0.86837572	-1.23311191	2.92E-07	-1.17837165	6.362E-07	NA	NA
	KCNJ11	0.83991577	0.01426651	0.03195187	0.98863756	-0.54237846	0.16515224	-0.88084167	0.00039333	NA	NA
	PKM	0.76444825	0.00302404	0.46498419	0.13642505	0.20150029	0.67026294	-0.180196	0.56185685	NA	NA

	PRKAA1	-0.32569348	0.34861841	-0.37780463	0.39576615	NA	NA	0.03030473	0.97520009	NA	NA
	PRKAA2	-0.17848726	0.77735126	-0.45581174	0.46821779	NA	NA	-0.54496368	0.10388739	NA	NA
	PRKAB1	0.32241473	0.51031823	0.43129954	0.37670528	NA	NA	0.15639583	0.7529501	NA	NA
	PRKAB2	-0.06003248	0.93225636	0.16124752	0.80076281	NA	NA	-0.00917674	1	NA	NA
	PRKAG1	-0.13660779	0.78184741	0.03896643	0.97872608	NA	NA	-0.0865219	0.88817857	NA	NA
	PRKAG2	-0.17587764	0.71744822	0.01995543	1	NA	NA	-0.1714283	0.72407064	NA	NA
	STK11	0.88139477	0.02770717	0.44391202	0.32461142	NA	NA	0.32564019	0.3695127	NA	NA
Olea	CAMKK1	0.72662135	0.08340932	0.25429222	0.63543234	NA	NA	-0.02867542	0.97768089	NA	NA
	CAMKK2	0.49081404	0.26657269	0.16062838	0.81382779	NA	NA	-0.07865222	0.91035231	NA	NA
	ACACA	0.50269274	0.13976161	-0.14886988	0.79322239	NA	NA	-0.05543691	0.93701432	NA	NA
	MTOR	0.13766349	0.7830213	-0.30203638	0.54495562	NA	NA	-0.16464596	0.73001695	NA	NA
	RPTOR	0.46255718	0.33943575	0.29400434	0.58303673	NA	NA	0.10637019	0.85271251	NA	NA
	ABCC8	0.53719553	0.06208706	-0.06227236	0.9320819	NA	NA	0.89177749	0.00094198	NA	NA
	KCNJ11	0.98962818	0.00290719	0.00863735	1	NA	NA	-0.19200731	0.65739058	NA	NA
	PKM	0.20150029	0.67026294	0.05208687	0.94618475	NA	NA	0.32786165	0.35226336	NA	NA
	PRKAA1	-0.22411603	0.58390952	0.02173497	0.93657663	NA	NA	NA	NA	NA	NA
Palm	PRKAA2	-0.03401781	0.99742847	0.32298732	0.35195577	NA	NA	NA	NA	NA	NA
	PRKAB1	0.59393194	0.15700488	0.1441175	0.63897398	NA	NA	NA	NA	NA	NA
	PRKAB2	-0.19041438	0.7064662	-0.1397653	0.61051808	NA	NA	NA	NA	NA	NA

	PRKAG1	0.16805774	0.74484738	-0.0240054	0.94066866	NA	NA	NA	NA	NA	NA
	PRKAG2	-0.01850741	1	-0.27781931	0.29634841	NA	NA	NA	NA	NA	NA
	STK11	0.86339405	0.0373708	0.09581526	1	NA	NA	NA	NA	NA	NA
	CAMKK1	0.69843509	0.10096924	-0.11217296	1	NA	NA	NA	NA	NA	NA
	CAMKK2	-0.07247567	0.95978383	-0.5089681	1	NA	NA	NA	NA	NA	NA
	ACACA	0.01495999	1	0.01050823	1	NA	NA	NA	NA	NA	NA
	MTOR	-0.23767433	0.62774477	-0.01613237	1	NA	NA	NA	NA	NA	NA
	RPTOR	0.19252387	0.8005774	0.53849852	1	NA	NA	NA	NA	NA	NA
	ABCC8	0.39951189	0.20869012	0.0349248	1	NA	NA	NA	NA	NA	NA
	KCNJ11	1.17995919	0.00035816	-0.00912754	1	NA	NA	NA	NA	NA	NA
	PKM	0.12153147	0.79519556	0.00989905	1	NA	NA	NA	NA	NA	NA
	PRKAA1	NA	NA	NA	NA	0.26369083	0.53113507	0.01388004	0.98206745	-0.10910477	0.92609992
	PRKAA2	NA	NA	NA	NA	-0.03378675	0.98748397	-0.94968973	0.00019985	0.0707493	0.96566766
	PRKAB1	NA	NA	NA	NA	0.19694752	0.68514387	0.65670676	0.01013924	-0.0613248	0.97062965
	PRKAB2	NA	NA	NA	NA	0.03506251	0.97983967	0.42123461	0.11498708	0.36185827	0.46227793
G25/Olea	PRKAG1	NA	NA	NA	NA	-0.16609006	0.75155842	0.57335126	0.0282347	-0.03619949	0.99429187
	PRKAG2	NA	NA	NA	NA	-0.15911301	0.78349281	-0.46465217	0.08976175	-0.07645001	0.95945937
	STK11	NA	NA	NA	NA	0.0029682	1	0.21903831	0.44322328	0.38295686	0.53459537
	CAMKK1	NA	NA	NA	NA	-0.81327663	0.01332054	-0.03561137	0.92831622	-0.48548432	0.24809036
	CAMKK2	NA	NA	NA	NA	0.41677359	0.27481412	-0.19705954	0.55049352	-0.00083108	1

	ACACA	NA	NA	NA	NA	-0.10081007	0.8864525	0.22031836	0.44322328	0.29660849	0.61196914
	MTOR	NA	NA	NA	NA	0.47297213	0.1814148	0.27644242	0.32786497	-0.00502049	1
	RPTOR	NA	NA	NA	NA	0.10355898	0.91799668	0.82405229	0.00089794	0.5691141	0.29607134
	ABCC8	NA	NA	NA	NA	-0.77224418	0.00366912	-0.57068411	0.02052841	-0.77224418	0.00366912
	KCNJ11	NA	NA	NA	NA	-0.71880581	0.03147494	-0.63019533	0.01102382	-0.44057531	0.34329796
	PKM	NA	NA	NA	NA	-0.02205608	1	-0.13098159	0.66671808	-0.02205608	1
G25/Palm	PRKAA1	NA	NA	NA	NA	0.19584549	0.64405476	NA	NA	-0.27533951	0.61758763
	PRKAA2	NA	NA	NA	NA	0.08467402	0.89321619	NA	NA	-0.07738671	0.96875907
	PRKAB1	NA	NA	NA	NA	0.12174617	0.81742555	NA	NA	0.04203216	0.99024952
	PRKAB2	NA	NA	NA	NA	0.22945029	0.58486287	NA	NA	-0.13712045	0.89305629
	PRKAG1	NA	NA	NA	NA	-0.31230448	0.38740765	NA	NA	-0.0057927	1
	PRKAG2	NA	NA	NA	NA	-0.19903085	0.67037095	NA	NA	-0.18771672	0.80981201
	STK11	NA	NA	NA	NA	-0.23887905	0.62503253	NA	NA	0.7315416	0.04744368
	CAMKK1	NA	NA	NA	NA	-1.19476563	4.28E-05	NA	NA	-1.16802015	7.52E-05
	CAMKK2	NA	NA	NA	NA	0.3818638	0.28455947	NA	NA	-0.35998198	0.59882575
	ACACA	NA	NA	NA	NA	-0.38368521	0.2855764	NA	NA	0.21175025	0.77572071
	MTOR	NA	NA	NA	NA	0.10355069	0.86082733	NA	NA	-0.02077685	1

RPTOR	NA	NA	NA	NA	-0.10015328	0.90237167	NA	NA	0.26931846	0.79490556
ABCC8	NA	NA	NA	NA	-2.05512599	4.22E-19	NA	NA	-0.32989688	0.45086671
KCNJ11	NA	NA	NA	NA	-0.97936027	0.00098898	NA	NA	0.17099569	0.85140359
PKM	NA	NA	NA	NA	0.05875529	0.92378936	NA	NA	-0.24258699	0.66454872

Supplementary Table S2: Clinical Data related to human islets

Donor	Gender	Age (years)	BMI (kg/m²)	Cause of death	Culture (day)	Viability (%)	Purity (%)
#1	M	56	28.6	Crania trauma	3	90	50
#2	M	59	27.2	Trauma	2	90	60
#3	F	41	22.4	Cerebral haemorrage	2	90	80
#4	M	46	27.2	Cerebral haemorrage	2	90	75
#5	F	59	23.7	Cerebral trauma	4	90	84
#6	M	49	26.2	Cerebral haemorrage	5	90	80
#7	M	39	22.9	Cerebral haemorrage	4	90	80
#8	M	56	22.5	Cerebral haemorrage	3	85	87

#9	M	60	29.1	Cerebral haemorrage	2	85	80
#10	M	34	24.7	Trauma	1	85	65
#11	M	50	22.2	Cerebral trauma	1	90	90
#12	F	53	26.0	Cerebral haemorrage	3	80	80
M (9)	%	49.9 ±9.0	25.6 ± 2.6		2.6 ±1.3	88.3 ±2.5	74.1 ±13.2
F (3)	%	51.0 ±9.2	24.0 ± 1.8		3.0 ±1.0	86.7 ± 5.8	81.3 ±2.3
(12)	100%	50.2 ±8.6	25.2 ± 2.5		2.7 ±1.2	87.9 ±3.3	75.9 ±11.7

Supplementary Table S3: Rat and human primers used for quantitative RT-PCR analysis

Species	Primer name	Sequence
**Rat**	*Ampk (Prkaa1)*	Fwd: 5’-AGT CAA AGC CGA CCC AAT GAC-3’<br>Rev: 5’-CTT CCT TCG CAC ACG CAA AT-3’
**Rat**	*Ampk (Prkaa2)*	Fwd: 5’-GAA GAT CGG ACA CTA CGT GCT-3’<br>Rev: 5’-TGC CAC TTT ATG GCC TGT CAA T-3’
**Rat**	*Ppia*<br>(cyclophilin)	Fwd: 5’-ATC TGC ACT GCC AAG ACT GA-3’<br>Rev: 5’-TCT TGC TGG TCT TGC CAT TC-3’
**Human**	*AMPK (PRKAA1)*	Fwd: 5’ -GGT GTA AGG AAA GCA AAA TGG C-3’<br>Rev: 5’-AGG ATT CTT CCT TCG TAC ACG-3’
**Human**	*AMPK (PRKAA2)*	Fwd: 5’- CAC ATG AAT GCC AAG ATA GCC G-3’<br>Rev: 5’-CTG CCT GAG ATG ACT TCA GGT GC-3’
**Human**	*PPIA*<br>(cyclophilin)	Fwd: 5’- ATC TGC ACT GCC AAG ACT GA -3’<br>Rev: 5’-TCT TGC TGG TCT TGC CAT TC -3’
